# Anthocyanin-Rich Blackcurrant Supplementation as a Nutraceutical Ergogenic Aid for Exercise Performance and Recovery: A Narrative Review

**DOI:** 10.1016/j.cdnut.2024.104523

**Published:** 2024-12-10

**Authors:** Mark ET Willems, Sam D Blacker, Stefano Montanari, Matthew D Cook

**Affiliations:** 1 Institute of Applied Sciences, University of Chichester, Chichester, United Kingdom; 2 Faculty of Arts, Science and Technology, University of Northampton, Northampton, United Kingdom; 3 School of Sport and Exercise Science, University of Worcester, Worcester, United Kingdom

**Keywords:** blackcurrant, anthocyanins, sport nutrition, exercise performance, exercise recovery

## Abstract

Athletes and physically active individuals consume sport nutrition supplements to enhance competitive sport performance and exercise recovery. Polyphenols have emerged as a promising area of research with application for sport and exercise nutrition owing to affecting physiologic mechanisms for exercise performance and recovery. The anthocyanin is a polyphenol that can be abundantly present in dark-colored fruits, berries, and vegetables. Anthocyanins and anthocyanin-induced metabolites will provide antioxidant and anti-inflammatory effects. The focus of this narrative review was on the observations with intake of anthocyanin-rich blackcurrant supplements on whole-body exercise performance and exercise recovery. This review included 17 studies with a randomized placebo-controlled crossover design (10 studies on performance and 8 on recovery effects) and 1 with a randomized placebo-controlled parallel group design (recovery effects). Among the performance studies, 6 studies (60%) reported positive effects, 3 studies (30%) reported no significant effects, and 1 study (10%) reported a mixed outcome. Among the recovery studies, 7 studies (78%) reported positive effects, 1 study (11%) reported no significant effects, and 1 study (11%) reported a negative effect. Studies with intake of supplements made from New Zealand blackcurrants (dose: 1.8–3.2 mg/kg and 105–315 mg anthocyanins, acute to 7-d intake) provided meaningful (but not always consistent) effects on continuous and intermittent exercise performance tasks (i.e. rowing, cycling, and running) and markers for exercise recovery. A mechanistic understanding for the beneficial exercise effects of anthocyanins for athletes and physically active individuals is still limited. Future work requires a better understanding of the specific types of anthocyanins and anthocyanin-induced metabolites and their effects on altering cell function that can enhance exercise performance and recovery.

## Introduction

Tissues of higher plants contain the flavonoid compound anthocyanins [1]. Anthocyanins are anthocyanidins aglycones with incorporation of 1 or more carbohydrates. The diversity in chemical structures of the anthocyanidins aglycones allowed the identification of hundreds of anthocyanins [2]. In higher plants, the anthocyanins contribute to the color variety of berries and fruits [2] and offer protection against environmental stresses [3]. For humans, the presence of the anthocyanins in the leaves, seeds, berries, and fruits make them readily available for consumption. Humans consume primarily the anthocyanidins such as cyanidin, delphinidin, malvidin, pelargonidin, peonidin, and petunidin [4]. In Europe, for example, intake of total anthocyanidins for men and women were 64.88 (SE 1.86) and 44.08 (SE 2.45) mg/d [4]. The evidence for the health-related effects of anthocyanin intake is growing, where benefits have been shown to include a reduced risk for cardiovascular disease and type 2 diabetes [[5], [6], [7]]. However, no dietary guidelines exist yet for the amount of intake of anthocyanins as part of a healthy diet. In fact, among the thousands of flavonoids, an intake recommendation of 400-600 mg/d only exists for flavan-3-ols, due to reported cardiometabolic health implications [8]. Existing and future research on the effects of the intake of anthocyanins in humans, with focus on beneficial health effects [9], will eventually provide the evidence to support intake recommendations for anthocyanins.

In blackcurrants, of the 15 anthocyanins present, cyanidin-3-*O*-glucoside, cyanidin-3-*O*-rutinoside, delphinidin-3-*O*-glucoside, and delphinidin-3-*O*-rutinoside make up >97% of the total anthocyanin content [10]. However, different fruits and berries have their own unique anthocyanin composition [11]. For anthocyanins, the low plasma bioavailability [12] strongly indicates that the numerous anthocyanin-induced metabolites are causally linked with providing the health effects [13]. The health-related effects of anthocyanin intake are thought to be due to antioxidant, anti-inflammatory, and immunomodulatory effects [14,15]. In support, the intake of anthocyanins (∼35 mg chokeberry anthocyanins daily for 4 wk) can enhance the endogenous antioxidant system [16]. However, note that the antioxidant capacity of the individual anthocyanins contributes to the total antioxidant capacity relative to the weight of the berry [11]. The potential of plant compounds to enhance the antioxidant capacity of human tissues as well as inhibition of the exercise-induced inflammatory response suggest the ergogenic potential for applications in sports and exercise. Such application has been shown, for example, for some of the flavan-3-ols in green tea extract with the ability to affect fat metabolism [17]. The ability of skeletal muscle tissue to enhance cellular antioxidant defense may limit exercise-induced fatigue [18] and enhance exercise performance [19]. However, it was only in the study by Matsumoto et al. [12] in which first observations were provided of fatigue-lowering responses in humans in a nonexercise study after intake of blackcurrant anthocyanins. Following an acute intake of 17 mg/kg body weight of blackcurrant anthocyanins, there was an increase in left forearm blood flow found using near infrared spectroscopy during typing activity, and there were changes in maximum total hemoglobin in the trapezius after maximal voluntary contractions after the typing [12]. In addition, an intake for 2 wk of 7.7 mg/kg body weight tended to lower the muscle activity (i.e. lower electromyographic root mean square) during intermittent typing work, suggestive of a reduction in muscle fatigue [12]. The observations by Matsumoto et al. [12] seemed not to have been recognized for some years, and only in the last decade, many studies have addressed the efficacy of blackcurrant anthocyanin intake on the physiologic, cardiovascular, and metabolic responses during exercise, as well as the potential to affect exercise recovery.

In this narrative review, the focus was on the studies that provided experimental evidence for the efficacy of anthocyanin-rich blackcurrant supplements (not whole food) to enhance whole-body exercise performance and recovery. Studies on effects in muscle groups will not be considered [20,21]. Studies with combined intake of supplements including anthocyanin-rich blackcurrant and conference proceedings will not be considered. For the purpose of this review, exercise performance is considered a task completion that may provide competitive advantage, for example, a faster running sprint time, cycling time-to-exhaustion or cycling time trial. Recovery is considered as the observable physiologic, metabolic, and functional phenomena after completion of an exercise task, so not solely an exercise performance task during the recovery phase. Animal and in vitro studies will only be considered for the purpose of our understanding of the potential causal mechanism by the intake of anthocyanins for the exercise performance and exercise recovery effects in humans. We will consider the strengths and the limitations of the experimental studies in humans and highlight the substantial gaps that still exist in our understanding of the optimal dosing strategy and the mechanisms for whole-body exercise performance and recovery effects by intake of anthocyanin-rich blackcurrant. As the first studies on the effects of anthocyanin intake examined postexercise effects, the review aimed to cover first the studies on recovery and subsequently the studies on the exercise performance effects. Observations from studies with clinical cohorts were covered when they allow insight into the mechanism for the exercise and recovery effects. It is not within the scope of this narrative review to provide detailed commentary on the metabolism of anthocyanin-induced metabolites and whole-body physiologic mechanisms underpinning the performance enhancing and exercise recovery effects. With the information available, the literature has provided meaningful observations with primarily intake of anthocyanin-rich supplementation made from New Zealand grown blackcurrant. New Zealand grown blackcurrant has higher concentrations of anthocyanins compared with non–New Zealand grown blackcurrant [22]. The available evidence obtained with studies on the effects of anthocyanin-rich blackcurrant will justify future work on the intake of a variety of anthocyanin-rich natural supplements to advance the field of sport and exercise nutrition [23,24].

## Early Evidence on the Effect of Intake of Anthocyanins on Exercise Recovery in Humans

The required time for the functional and physiologic recovery from exercise-induced effects, such as structural muscle damage, muscle soreness, muscle dysfunction, oxidative stress, and inflammation, can take days to weeks. The occurrence of these exercise-induced effects will depend largely on the intensity and duration of the exercise, particularly when the exercise was of an unaccustomed nature [25,26]. In a physical exercise training program, the exercise-induced oxidative stress and the inflammatory response postexercise contribute to the physiologic adaptation processes [27,28]. In addition, adaptations from physical training can include an enhanced endogenous antioxidant system [29]. Therefore, regular intake of polyphenols (including anthocyanins) in the diet is a nonexercise nutritional means for enhancing the endogenous antioxidant system [30]. An enhanced endogenous antioxidant system is beneficial for coping with exercise-induced oxidative stress. One of the first studies to examine the effects of chronic intake of anthocyanins on exercise-induced oxidative stress was with chokeberry juice [16]. Chokeberry juice is composed of ≤98.7% of cyanidin glycosides and consist of cyanidin-3-*O*-galactoside, cyanidin-3-*O-*glucoside, cyanidin-3-*O*-arabinoside, and cyanidin-3-*O*-xyloside [31]. Pilaczynska-Szczesniak et al. [16] dosed 19 male elite rowers (members of the Polish rowing team) with chokeberry (*Aronia melanocarpa L.*) juice (3 × 50 mL/d) for 4 wk. The chokeberry juice in the study contained 23 mg anthocyanins per 100 mL. Four weeks intake of the chokeberry juice reduced concentrations of thiobarbituric acid reactive substances, a measure of lipid peroxidation, 1 min and 24 h postexercise following an incremental rowing protocol, providing support for enhancement of the antioxidant defense system and therefore enhancement recovery [16]. The enhanced endogenous antioxidant system by polyphenol intake or exercise is due to the Kelch-like ECH-associated protein 1-nuclear factor erythroid 2-related factor 2 system [32,33]. Evidence for the activation of this system by chokeberry, however, remains absent from the literature but is present, for example, for raspberry (primarily containing cyandin-3-*O*-glucoside) in human HepG2 cells [34]. It is surprising that considering the dark blue–colored chokeberry to be a very rich source of polyphenols with anthocyanins [35], no studies with chokeberry intake in humans have provided exercise performance-enhancing effects [36]. It is possible that some anthocyanin-rich berries may provide beneficial effects for exercise recovery without a performance-enhancing effect. One of the key messages of the present review is, therefore, that future work needs to address the specificity of the anthocyanins or combination of anthocyanins, which can be effective for both exercise recovery and performance or those single or combination of anthocyanins that only seem to have either recovery or performance effects. Studies with such aims have not been considered or started in humans. In the C57BL/6 mouse model of polygenic obesity, for example, diets that were supplemented with different berries provided different metabolic effects, such as differences in both mitochondrial respiration and the dissipation of the mitochondrial proton gradient in adipose tissue [37], and fasting glucose [38]. Studies in humans on the effects of single anthocyanins on physiologic, metabolic, cardiovascular, and exercise performance responses are absent. In addition, many reviews on the effects of polyphenols in humans have not clearly recognized the importance of the potential differences in effects by intake of different combinations of anthocyanins [39,40]. In the following section, we will consider the evidence for the intake of anthocyanin-rich blackcurrant for exercise recovery.

## Anthocyanin-Rich Blackcurrant and Exercise Recovery

The recovery from the occurrence of exercise-induced muscle fatigue and potential muscle injury of habitual exercise training sessions will require normally not more than a few days. Athletes in preparation for competitive endurance events (e.g. marathon running) will have adopted tapering strategies to optimize recovery from the progression in training volume. However, during physical preparatory training or busy competitive schedules, an enhanced exercise recovery would benefit the athletes for undertaking subsequent exercise training sessions and competition. Studies with bioactive nutritional compounds that have antioxidant and anti-inflammatory properties provided experimental evidence to accelerate the recovery from exercise-induced muscle fatigue and muscle injury. Early studies focused on effects of antioxidant delivering ability of vitamins E and C [41]. More recently, strong and abundant experimental support has emerged for the effectiveness of anthocyanin-containing tart cherry supplementation on exercise recovery providing anti-inflammatory effects. Tart cherry contains primarily the anthocyanins cyanidin-3-glucosylrutinoside and cyanidin-3-rutinoside, that is ∼ 42% and 35% of total anthocyanins, respectively [42]. In a systematic review and meta-analysis on tart cherry supplementation [43], small beneficial effects were reported after strenuous exercise for muscle soreness (effect size: −0.44); sprint time (effect size: −0.32); C-reactive protein (effect size: −0.46) and IL-6 (effect size: −0.35); moderate effects for muscle strength (effect size: −0.78) and muscular power (effect size: −0.53); and large effects for jump height (effect size: −0.82).

In studies on the effects of intake of blackcurrant on exercise recovery, meaningful effects have been reported for the recovery from different exercise modalities that had variation for exercise duration and intensity (Table 1) [[44], [45], [46], [47], [48], [49], [50], [51], [52]]. As far as we know, the first study to examine the exercise recovery effects with intake of blackcurrant was done by the New Zealand Institute for Plant and Food Research, Ltd [44]. Lyall et al. [44] examined the exercise recovery effects by the intake of 120 mg blackcurrant anthocyanins before and after 30 min of indoor rowing at 80% maximum oxygen uptake ($\dot{V}$O_2MAX_) in a recreationally active cohort (5 females, 5 males) with a broad age range (37–63 y; mean ± SD: 48 ± 3 y). Blackcurrant lowered protein carbonyl concentrations 30-min after exercise that were normally elevated by ∼40% by the indoor rowing at 80% $\dot{V}$O_2MAX_ and indicative of oxidative stress. The effect was of short duration as blackcurrant had no effect on plasma protein carbonyl concentrations at 1, 2, and 24 h during recovery, but observations on IL-6, TNFα and creatine kinase were indicative of lower inflammation and reduced muscle damage. In the study by Lyall et al. [44], the dosing strategy was not justified. However, based on the dosing information, intake of 120 mg blackcurrant extract was effective within 30 min, suggesting not only fast bioavailability of anthocyanins and anthocyanin-induced metabolites—for example, Costello et al. [53] for presence over 6 h and Czank et al. [54] for 48 h—but maybe also rapid physiological antioxidative stress effects. However, the potential effects of the many metabolites, for example, protocatechuic acid, vanillic acid, and gallic acid [53,54], on the alteration of in vivo cell function are not known. In addition, Czank et al. [54] identified 24 metabolites and observed substantial interindividual differences in recovery rate (15.1%–99.3%) of 25 ^13^C-labeled compounds that consisted of ^13^C_5_-cyanidin-3-glucoside and the 24 labeled metabolites. The distribution, metabolism, and excretion of the anthocyanin-induced metabolites is beyond the focus of this narrative review. As far as we know, no information is available on the plasma availability of anthocyanins and parent metabolites by different intake strategies of blackcurrant over a long period; see Kalt et al. [55] for a study with intake of blueberry juice. In addition, more understanding is needed for the role of the gut microbiome on the plasma availability of anthocyanins and parent metabolites [56,57]. In a subsequent study by the New Zealand Institute for Plant and Food Research, Ltd, 30 min of indoor rowing at 70% $\dot{V}$O_2max_ in healthy individuals reduced plasma protein carbonyl concentrations not at 0 h but 2 h during recovery (compared with 0 h) with 0.8 and 1.6 mg/kg blackcurrant [45]. Thus, studies show inconsistent observations on plasma protein carbonyl concentrations as a marker of oxidative stress after exercise with intake of blackcurrant [44,45]. Another marker of oxidative stress, the lipid oxidation product malondialdehyde, was reduced 2 h after 30 min of indoor rowing at 70% $\dot{V}$O_2max_ in healthy individuals with similar efficacy with acute (1 h before) and 5 wk daily intake of 3.2 mg/kg (∼240 mg) of blackcurrant anthocyanins [46]. In addition to the evidence of enhanced recovery with markers of oxidative stress, Lyall et al. [44] also observed blunting of the rise in creatine kinase concentrations 24 h after exercise, an indication that blackcurrant affects the symptoms of exercise-induced muscle damage. This was also observed in a study by Hunt et al. [47] in which 12 d of daily intake of 105 mg blackcurrant anthocyanins in extract form (8 d before and 4 d after exercise) reduced muscle soreness and creatine kinase concentrations during the recovery from 4 × 15 maximal concentric and eccentric contractions of the biceps brachii muscle. However, no enhanced functional recovery was shown in Hunt et al. [47]. In contrast, muscle soreness and countermovement jump variables were not affected by intake of blackcurrant anthocyanins (7 d before and 2 d after with 210 mg/d anthocyanins) following a half-marathon run in recreationally runners (*n* = 20, 8 females) [48]. In addition, no effect on muscle soreness was reported after eccentric squatting in healthy participants (*n* = 24, 6 females) with intake of blackcurrant nectar (2× per day for 8 d with ∼370 mg/d anthocyanins) but with lower creatine kinase responses [49]. In Perkins et al. [50], the absolute lactate concentrations declined faster during recovery from an intermittent high-intensity running protocol in recreationally active male team sport players following a 7-d intake of 105 mg blackcurrant anthocyanins in extract form. Faster decline for absolute lactate concentrations during recovery was also shown in the 20-min after completion of a 16.1-km time trial [51]. However, the recovery phase in the blackcurrant conditions in Perkins et al. [50] and Cook et al. [51] were initiated with higher lactate concentrations, suggesting a potential role for the mass action effect. In a cycling study by Murphy et al. [52], the time to complete a 4-km time trial following 10 min of active recovery after a 4-km time trial was not enhanced. With the information available, studies on effects of New Zealand blackcurrant (NZBC) extract have not provided convincing evidence for enhanced endurance performance during a short exercise recovery period. In addition, it remains unclear whether the potential enhanced exercise recovery by intake of NZBC is beneficial for nonendurance exercise tasks. Athletes will only benefit from enhanced exercise recovery when it would enhance subsequent competitive sport performance or allowing enhanced adaptation from subsequent physical training sessions. In the next section, we will focus on the effects of intake of blackcurrant on whole-body exercise performance, that is, continuous endurance exercise and intermittent exercise.

**TABLE 1:** Summary of studies in humans examining the effects of intake of blackcurrant anthocyanins on exercise recovery.

Source	Cohort	Design	Dosing strategy	Exercise task	Primary recovery outcomes with NZBC
Lyall et al., 2009 [44]	5 females, 5 males, age: 48 ± 2.5 y, recreationally active	Double-blind, placebo-controlled, randomized, crossover	240 mg NZBC anthocyanins (120 mg before and after exercise)	30 min of rowing at 80% ${\dot{V}O}_{2\;\max}$	↓ Plasma carbonyl concentrations (0 h postexercise); ↓ creatine kinase (24 h postexercise)
Hurst et al., 2019 [45]	Healthy individuals, *n* = 32, age range: 20–60 y	Double-blind, placebo-controlled, randomized, parallel	1 × 0.8, 1.6, or 3.2 mg/kg of NZBC anthocyanins	30 min of rowing at 70% ${\dot{V}O}_{2\;\max}$	Lower postexercise (0 h) heart rate with 0.8 and 1.6 mg/kg blackcurrant, no effect on postexercise lactate, ↓ plasma carbonyl concentrations (2 h postexercise with 0.8 and 1.6 mg/kg blackcurrant
Hurst et al., 2020 [46]	Healthy individuals, *n* = 32, age range: 20–60 y	Double-blind, placebo-controlled, randomized, parallel	Acute (1 h before) and 5 wk daily intake of 3.2 mg/kg (∼240 mg) of NZBC anthocyanins	30 min of indoor rowing at 70% ${\dot{V}O}_{2\;\max}$	↓ Malondialdehyde 2 h postexercise with acute and chronic intake
Hunt et al., 2021 [47]	Healthy nonresistance trained individuals, NZBC: *n* = 14, 10 women, age: 24 ± 2 y); placebo: *n* = 13, 9 women, age: 23 ± 2 y	Double-blind, placebo-controlled, randomized, parallel	Each day 105 mg NZBC anthocyanins in extract form for 12 d	4 × 15 repetitions of maximal concentric and eccentric contractions of the biceps brachii muscle in the dominant arm	↓ Muscle soreness at 24 and 48 h postexercise; ↓ creatine kinase at 96 h postexercise
Costello et al., 2020 [48]	Healthy individuals, NZBC: *n* = 10, 4 women, age: 30 ± 4 y; placebo: *n* = 10, 4 women, age: 29 ± 7 y	Double-blind, placebo-controlled, randomized, parallel	Each day 210 mg NZBC anthocyanins in extract form for 9 d	Outdoor half-marathon race	↑ Urine IL-6 at 48 h postexercise
Hutchison et al., 2016 [49]	Healthy individuals, blackcurrant: *n* = 8, 7 women, age: 19.5 ± 0.3 y; placebo: *n* = 8, 6 women, age: 20.9 ± 0.9 y	Double-blind, placebo-controlled, randomized, parallel	Each day 2× ∼369 mg blackcurrant anthocyanins (country unknown) in drinks for 8 d	Eccentric knee extensions (3 × 10 sets at 115% of 1-repetition maximum	↓ Creatine kinase at 48 and 96 h postexercise; ↓ plasma IL-6 concentrations at 24 h postexercise)
Perkins et al., 2015 [50]	13 men, recreationally active, age: 25 ± 4 y	Double-blind, placebo-controlled, randomized, crossover	105 mg NZBC anthocyanins in extract form for 7 d	Progressive high-intensity, intermittent treadmill running	Faster decline in blood lactate for 30 min postexercise
Cook et al., 2015 [51]	14 men, cycling 8–10 h/wk, age: 38 ± 13 y	Double-blind, placebo-controlled, randomized, crossover	105 mg NZBC anthocyanins in extract form for 7 d	16.1-km cycling time trial	Faster decline in blood lactate for 20 min postexercise
Murphy et al., 2017 [52]	10 men, cyclists, age: 30 ± 12 y	Double-blind, placebo-controlled, randomized, crossover	105 mg NZBC anthocyanins, in extract form for 7 d	2 × 4-km cycling time trial with 10 min in between with active recovery	No change in time for the second 4-km cycling time trial

NZBC, New Zealand blackcurrant; $\dot{V}$O_2MAX_, maximum oxygen uptake.

## Anthocyanin-Rich Blackcurrant and Exercise Performance

Exercise performance is dependent upon multiple intrinsic (e.g. physiologic and morphologic components) and extrinsic factors (e.g. motivation, sleep, and nutritional practices) [58]. Competitive athletes are keen consumers of performance-enhancing nutritional ergogenic aids [59]. The performance-enhancing effects by the traditional supplements caffeine, creatine, β-alanine, sodium bicarbonate, dietary nitrate, and glycerol are recognized [60], with the enhancing effects related to the intensity and duration of the exercise approach—for example, for a review on caffeine, see [61]. Anthocyanins have emerged as a performance-enhancing nutritional aid [24]. This may be linked with the known potency to scavenge active oxygen species, with first in vivo evidence in the liver of rats [62]. At that time, there was also the concern for a role for reactive oxygen species affecting mechanisms to provide higher peripheral muscle fatigue [63]. However, it needs to be noted that there is no convincing evidence for an antioxidant free radical scavenging effect by intake of anthocyanins in in vivo contracting human skeletal muscle. Nevertheless, postponing in a meaningful way the peripheral (and central) fatigue mechanisms by physical training modalities or different traditional and nontraditional nutritional supplements (e.g. blackcurrant supplementation) has the potential to enhance exercise performance.

## Whole-Body Continuous Endurance Exercise

Many studies, primarily from the University of Chichester (United Kingdom), have shown enhanced exercise performance effects for a variety of exercise tasks but with some inconsistency in the findings (Table 2) [51,[64], [65], [66], [67], [68]]. In the study by Cook et al. [51], first observations were provided for the effectiveness of NZBC extract (7 × 105 mg/d anthocyanins) on a 16.1-km ergometer cycling time trial in trained male cyclists (*n* = 14, $\dot{V}$O_2MAX_: 53 ± 6 mL/kg/min). To be included in the study, participants had to cycle 8–10 h/wk in addition to having a personal best time for a 16.1-km under 30 min. The nonpaid cohort of trained male cyclists in the study by Cook et al. [51] had 2 full familiarizations as part of the study. Full familiarization is a methodologic necessity that is required to minimize or exclude task learning effects and even more essential for participants unfamiliar with an exercise task [69]. More than 1 full familiarization may be essential to avoid the variation in the performance of unfamiliar tasks even for a trained cohort. In the study by Cook et al. [51], 11 participants had an increase in 16.1-km cycling time trial performance with a cohort observation of an increase in time of 2.4% (placebo: 1722 ± 131 s, NZBC extract: 1678 ± 108 s). The enhanced performance time of 2.4% was similar to what has been observed with acute intake of beetroot juice (0.5 L with 6.2 mmol of nitrate) for a cycling time trail of 16.1 km (i.e. 2.7% increase) in competitive male cyclists (*n* = 9, $\dot{V}$O_2MAX_: 56.0 ± 5.7 mL/kg/min) [70]. The study by Lansley et al. [70] also had 2 full familiarizations. A higher enhanced 15 km cycling performance time of 4.6% was observed with 6 d intake of Montmorency cherry powder (257 mg/d anthocyanins) in trained male cyclists (*n* = 8 and 2 full familiarizations) [71]. The early work with 7-d dosing with NZBC extract raised the question whether there may be an effect by the last dose on the day of testing. Recent work on the dosing time before exercise with Montmorency cherry indicated that ∼90 min before was better for enhanced 15-km cycling time trial performance than ∼30 and ∼150 min before [72]. In the study by Montanari et al. [64], an acute intake of 315 mg anthocyanins in male and female cyclists (fully familiarized 2 times, *n* = 34, $\dot{V}$O_2MAX_: 57 ± 5 mL/kg/min, 8 females) 120 min before enhanced performance for the 16.1-km cycling time trial, but only in the slower cyclists (i.e. >1400 s during the familiarizations). It is possible that the 80% positive response rate to intake of blackcurrant in the study by Cook et al. [51] was related to the cohort being considered slow cyclists according to Montanari et al. [64]. It needs to be noted also that the study by Montanari et al. [64] was a home-based study during COVID lockdown in which participants were informed that the acute 16.1-km cycling performance effects of 2 different products were tested. This approach is uncommon in sport nutrition research in which a randomized, placebo-controlled, crossover design remains the gold standard. Nevertheless, the study by Montanari et al. [64] evidenced that, for some athletes, beneficial cycling performance-enhancing effects can be obtained with acute intake of NZBC extract. Interestingly, a role for aerobic fitness level was also shown for nitrate loading with lower increases in plasma nitrate in those with high $\dot{V}$O_2PEAK_ and less improvement in 3-km field running time trial [73]. Therefore, we cannot exclude that anthocyanin-induced metabolite availability by intake of NZBC contributes to interindividual responses (and potentially nonresponses) in enhancement of exercise performance.

**TABLE 2:** Summary of studies in humans examining the effects of intake of blackcurrant anthocyanins on whole-body continuous exercise performance.

Source	Cohort	Design	Intake	Exercise task	Main outcomes
Cook et al., 2015 [51]	14 men, cycling 8–10 h/wk, age: 38 ± 13 y	Double-blind, placebo-controlled, randomized, crossover	105 mg NZBC anthocyanins in extract form for 7 d	16.1-km cycling time trial (laboratory testing)	2.4% faster time
Montanari et al., 2023 [64]	34 cyclists (8 women), age: 38 ± 7 y	Double-blind, placebo-controlled, randomized, crossover with participants informed that 2 different blackcurrant products were tested	Acute intake of 315 NZBC anthocyanins in extract form	16.1-km cycling time trial (home-based testing)	1.3% faster time in slower cyclist (>1400 s for 16.1 km)
Moss et al., 2023 [65]	Trained male runners, *n* = 16, age: 26 ± 5 y	Double-blind, placebo-controlled, randomized, crossover	Acute intake of 315 NZBC anthocyanins in extract form	5-km treadmill running	2.7% faster time
Montanari et al., 2020 [66]	13 male cyclists, age: 39 ± 10 y	Double-blind, placebo-controlled, randomized, crossover with dose effects	105 and 210 mg NZBC anthocyanins in extract form for 7 d with testing on day 1, 4, and 7	16.1-km cycling time trial (laboratory testing)	Inconsistent effects with 210 mg NZBC anthocyanins
Pastellidou et al., 2021 [67]	15 males, recreationally active, age: 24.4 ± 3.6 y	Double-blind, placebo-controlled, randomized, crossover	315 mg NZBC anthocyanins extract form for 2 d	Treadmill running to exhaustion at 110% of $\dot{V}$O_2max_	No cohort effect
Willems et al., 2019 [68]	11 male cyclists, age: 38 ± 11 y	Double-blind, placebo-controlled, randomized, crossover	210 mg NZBC anthocyanins extract form for 7 d	16.1-km cycling time trial (laboratory testing) in normobaric hypoxia	No cohort effect

NZBC, New Zealand blackcurrant; $\dot{V}$O_2MAX_, maximum oxygen uptake.

In trained male runners (*n* = 16, $\dot{V}$O_2MAX_: 55 ± 6 mL/kg/min), an acute intake of 315 mg anthocyanin-rich NZBC extract enhanced 5-km treadmill running performance by 2.8% (placebo time: 1346.33 ± 124.44 s) with 88% of the participants responding [65]. Future work is required to examine whether higher acute dosing is required to enhance the overall response rate in performance studies. In addition, it is not known whether prolonged dosing is required for the faster cyclists (<1400 s for a 16.1-km cycling time trial) in the study by Montanari et al. [64] and whether a dosing strategy for anthocyanin-rich NZBC can exist that provides a 100% meaningful response for performance enhancement in all participants. For caffeine, for example, a role for genetics is apparent for the interindividual response on cycling time trial performance [74]. For the ability of anthocyanins to enhance exercise performance, there is no research on the potential role of genetics to explain interindividual differences by intake of blackcurrant anthocyanins—see Miranda-Vilela et al. [75] for effects of gene polymorphisms on response to intake of pequi-oil supplementation. Interestingly, a study on the effects of pomegranate in patients with biochemical recurrence following treatment for prostate cancer showed ability to respond in those with the manganese superoxide dismutase (a mitochondrial antioxidant enzyme) genotype [76]. However, nongenotype factors will also contribute to variability of responses. For example, in a study on the dose (105 and 210 mg blackcurrant anthocyanins) and time (acute and 4 and 7 d) response to intake of NZBC extract the response for performance-enhancing effects for the 16.1-km cycling time trial was inconsistent [66]. For this study, 11 visits were required including washout periods of ≥2 wk resulting in completion times from 3 to 11 mo [66]. It is possible that the period to complete all testing allowed unwanted variation due to changes in seasonal activity lifestyles of the participants. In addition, the study had consistent morning testing for only 3 of the 13 participants [66]. In the study by Boyett et al. [77], trained male cyclists (*n* = 7) responded with a higher performance effect during morning testing for a 3-km cycling time trial than that during evening testing with intake of caffeine (6 mg/kg body weight). Future studies should address whether the time-of-day can affect the performance-enhancing potential of anthocyanin-rich NZBC for trained athletes. In addition, the observations on potential time-of-day effects would also inform our knowledge on the optimal dosing strategy required for time-of-day competitive athletic events. In another running study, Pastellidou et al. [67] examined the effect of NZBC extract (315 mg anthocyanins with 1-d preloading) on time-to-exhaustion running test at 2 different intensities in recreationally active males (*n* = 15, $\dot{V}$O_2MAX_: 53.1 ± 3.4 mL/kg/min). Time-to-exhaustion exercise performance tests compared with time trials are known to have high variation (i.e. >10%) [78]. Nevertheless, 2 time-to-exhaustion tests, one at critical speed (finish time: 1540–2284 s) and another at 110% of $\dot{V}$O_2MAX_ (185–367 s) provided no cohort effects, but the authors suggested a performance-enhancing effect for 60% the participants by 10%–20% [67]. Only 1 study examined the endurance performance-enhancing potential of intake of 7 d of NZBC extract (210 mg/d) in a stressful environmental condition (normobaric hypoxia: ∼15% of oxygen; i.e. ∼2500 m) and found no effect in trained male cyclists (*n* = 11, age: 38 ± 11 y, $\dot{V}$O_2MAX_: 47 ± 5 mL/kg/min) for a 16.1-km time trial [68]. In addition, in trained male cyclists (*n* = 10), there was not convincing evidence for a performance-enhancing effect (7 d intake of 105 mg/d anthocyanins) for a 4-km time trial [52]. It is possible that the study was underpowered. However, when a second 4-km time trail was performed, the total time for the two 4-km time trails was 0.82% faster [52].

There is an absence of exercise performance studies with female cohorts with intake of anthocyanin-rich blackcurrant; see Smith et al. [60] for a call for inclusion of females in sport nutrition studies. In the only study with performance testing of an outdoor 5-km run in trained female runners (*n* = 23, age: 31 ± 8 y, $\dot{V}$O_2max_: 49 ± 4 mL/kg/min), the intake daily over 3 wk in a training block was a fruit drink concentrate (of unknown composition) mixed with blackcurrant extract providing 300 mg anthocyanins and 15 mg vitamin C [79].

In summary, there is limited and not always consistent evidence for an endurance performance-enhancing effect of intake of NZBC. The available observations seem to indicate that there is no effectiveness for performance enhancement for relatively short, continuous, best-effort endurance tasks. More work is needed on the required dosing strategies (dose and time) with NZBC for enhancing endurance performance in specific female and male cohorts with different athletic endurance abilities (e.g. 5 km and half-marathon runners) and various endurance exercise modalities (e.g. rowing and cross-country skiing). In addition, future work is recommended to examine performance enhancement by intake of NZBC in combination with metabolomic approaches [80], which may support the understanding of interindividual responses.

## Whole-Body Intermittent Exercise

Many team sports require repeated high-intensity and maximal-intensity bouts of exercise, such as jumping in volleyball and repeated running sprints in football, field hockey, and rugby sevens [81,82]. The exercise-induced oxidative and inflammatory stress during repeated running sprints is probably higher than the oxidative stress during continuous exercise—for a role of exercise intensity on oxidative stress, see [83]. The first study on the effects on repeated and incremental high-intensity exercise by Perkins et al. [50] showed substantial changes in treadmill running performance with 7-d intake of NZBC extract (105 mg/d anthocyanins) in participants with experience in sports with high-intensity intermittent exercise (Table 3) [50,[84], [85], [86]]. In short, participants performed stages with 6 repeated high-intensity treadmill running bouts of 19 s each (starting speed at 80% of the running speed at $\dot{V}$O_2MAX_) with an active recovery of 15 s and passive recovery of 60 s between the incremental stages to the point of voluntary exhaustion [50]. The protocol used in Perkins et al. [50] was adapted from Mukherjee and Chia [87] for the testing of running capability in soccer players. For the cohort, the speed of the first running bout was 11.5 ± 5.7 km/h and the speed at exhaustion was 18.0 ± 1.2 km/h, with active recovery speeds 7.2 ± 3.6 km/h. With the intake of NZBC extract, participants were able to have a larger number of running bouts (placebo: 32 ± 4, blackcurrant: 35 ± 6; *P* = 0.02). In addition, the distance that was covered during the high-intensity treadmill running bouts was increased by 10.8% (placebo: 2572 ± 421 m; blackcurrant: 2849 ± 570 m; *P* = 0.024). Such effectiveness for NZBC extract for high-intensity treadmill running bouts justifies future work whether there is application in real-world competitive team sport settings [88], in addition to providing meaningful performance effects in elite athletes [89].

**TABLE 3:** Summary of studies in humans examining the effects of intake of blackcurrant anthocyanins on whole-body intermittent exercise with different intensities high-intensity and maximal-intensity exercise performance.

Source	Cohort	Design	Intake	Exercise task	Main outcomes
Perkins et al., 2015 [50]	13 men, recreationally active, age: 25 ± 4 y	Double-blind, placebo-controlled, randomized, crossover	105 mg NZBC anthocyanins in extract form for 7 d	Progressive high-intensity, intermittent treadmill running	Increase in number of high-intensity running bouts
Willems et al., 2016 [84]	13 men, recreationally active, age: 22 ± 1 y	Double-blind, placebo-controlled, randomized, crossover	105 mg NZBC anthocyanins in extract form for 7 d	Loughborough intermittent shuttle test	Reduced slowing of the 20-m maximal sprint in the final 15-min block
Godwin et al., 2017 [85]	Football players, recreationally active: *n* = 15, age: 20 ± 1 y; academy (professional club): *n* = 9, age: 17 ± 1 y	Double-blind, placebo-controlled, randomized, crossover	210 mg NZBC anthocyanins in extract form for 7 d	Running-based anaerobic sprint test	Reduced slowing of sprint 5 in academy players
Paton et al., 2022 [86]	12 well-trained male cyclists, age: 39.5 ± 11.4 y	Double-blind, placebo-controlled, randomized, crossover	Acute blackcurrant drink intake (300 mL containing 155 mg anthocyanins, 80 mg l-theanine, 50 mg pine bark extract, 7.7 g carbohydrate)	8 × 5-min maximal cycling	No effect

NZBC, New Zealand blackcurrant.

The effect of NZBC extract (7 d of 105 mg/d anthocyanins) was also examined in the performance of the Loughborough intermittent shuttle test (LIST) [84], a test mimicking also the physical demands of football. The LIST consists of 5 × 15-min blocks with distance running at set speeds and 9–10 maximal 20-m sprints. It was only in the final 2 blocks (i.e. after 45 min of exercise) that the effectiveness of NZBC extract became apparent with reduced slowing of the maximal sprint times [84]. This may indicate that a level of fatigue—see Daab et al. [90] for peripheral and central fatigue during the LIST—is required for meaningful effectiveness of intake of NZBC to enhance exercise performance. However, on completion of the 5 × 15-min blocks, no effect was observed for a time-to-exhaustion running task [84]. This may indicate that the effectiveness of NZBC extract to enhance exercise performance in a fatigued state may be task-dependent. Studies by Godwin et al. [85] and Potter et al. [91] also seem to confirm that some level of fatigue is required for NZBC to enhance exercise performance. NZBC extract (7-d intake of 210 mg/d anthocyanins) became only effective after the fourth sprint in trained youth football players during the running-based anaerobic sprint test. However, in the same study, NZBC extract had no effect in recreationally active football players during the running-based anaerobic sprint test [85]. In the study by Potter et al. [91], a trend (*P* = 0.062) for a larger hang time and enhancement of sport climbing performance was reported but potential methodologic and analytical issues were raised [92].

As far as we know, the only cycling study with intermittent exercise was performed by Paton et al. [86] in which effects of acute blackcurrant drink intake (300 mL containing 155 mg anthocyanins, 80 mg L-theanine, 50 mg pine bark extract, 7.7 g carbohydrate) were examined in well-trained male cyclists (*n* = 12, age: 39.5 ± 11.4 y, $\dot{V}$O_2PEAK_: 4.71 ± 0.61 L/min) for 8 × 5 min bouts of maximal cycling (with 2 min active recovery). No effects were observed for the blackcurrant drink [86]. It is possible that a higher acute dosage or chronic dosing is required to obtain effectiveness, as was observed by Montanari et al. [64], albeit for the 16.1-km cycling performance in faster cyclists. Nevertheless, Paton et al. [86] was the first study as well to examine acute effects by intake of an anthocyanin-rich blackcurrant drink during high-intensity intermittent exercise. Therefore, more work is required to optimize the dosing strategy for acute intake of blackcurrant anthocyanins to affect intermittent exercise performance.

## Conclusions and Future Directions

Intake of anthocyanins in the studies with extracts and powder made from NZBC have provided meaningful effects for whole-body exercise performance for continuous and intermittent exercise models and exercise recovery. Fruit-derived polyphenols are now recognized by the Australian Sports Commission with emerging potential as a performance-enhancing nutritional aid. However, the sheer diversity of berry-derived and fruit-derived polyphenols including the numerous anthocyanins will provide the academic community with an enormous challenge to establish optimal evidence-based dosing strategies in sport nutrition. Many anthocyanin-rich berries, and therefore many combinations of naturally existing anthocyanins, remain unexplored regarding the efficacy for recreationally active individuals and athletes. Dosing studies with manufactured single or specific multiple anthocyanins with application for athletes are likely to remain too costly to examine for effectiveness, at least in the short-term. It is expected that it will take decades for the field of anthocyanin in application for sport nutrition to provide the evidence on the dose and optimal combination of specific anthocyanins to establish maximal efficacy for exercise performance and recovery. The question remains whether the blackcurrant anthocyanins cyanidin and delphinidin have established themselves as a winning combination with application for sport nutrition. Time will tell! Future work on the efficacy of supplements with natural combination of berry anthocyanins is warranted to establish its potential and usefulness for the contribution of natural berry and fruit sources as an ergogenic aid in sport and exercise nutrition. In addition, future work should address also the efficacy and safety of combined intake of long-term intake of anthocyanins and other recognized sport nutrition supplements.

## Author contributions

The authors’ responsibilities were as follows — METW, SDB, SM, MDC: wrote the paper; METW: had primary responsibility for final content; and all authors: read and approved the final manuscript.

## Data availability

Data described in the manuscript is available from the published sources considered in the narrative review.

## Funding

Health Currancy Ltd, Blackcurrant New Zealand, Sujon (New Zealand), and the University of Chichester provided support for open access. The supporting sources were not involved in the content of the manuscript.

## Conflict of interest

The authors report no conflicts of interest.
